# Does Playground Improvement Increase Physical Activity among Children? A Quasi-Experimental Study of a Natural Experiment

**DOI:** 10.1155/2013/109841

**Published:** 2013-06-13

**Authors:** Erika E. Bohn-Goldbaum, Philayrath Phongsavan, Dafna Merom, Kris Rogers, Venugopal Kamalesh, Adrian E. Bauman

**Affiliations:** ^1^Prevention Research Collaboration, School of Public Health, The University of Sydney, NSW 2006, Australia; ^2^School of Science and Health, University of Western Sydney, Camperdown Campus, NSW, Australia; ^3^IMPACT Strategic Research Centre, School of Medicine, Deakin University, VIC, Australia

## Abstract

Outdoor recreational spaces have the potential to increase physical activity. This study used a quasi-experimental evaluation design to determine how a playground renovation impacts usage and physical activity of children and whether the visitations correlate with children's physical activity levels and parental impressions of the playground. Observational data and intercept interviews were collected simultaneously on park use and park-based activity among playground visitors at pre- and postrenovation at an intervention and a comparison park during three 2-hour periods each day over two weeks. No detectable difference in use between parks was observed at followup. In the intervention park, attendance increased among boys, but decreased among girls although this (nonsignificant) decline was less marked than in the comparison park. Following renovation, there was no detectable difference between parks in the number of children engaged in MVPA (interaction between park and time: *P* = 0.73). At the intervention park, there was a significant decline in girls engaging in MVPA at followup (*P* = 0.04). Usage was correlated with parental/carer perceptions of playground features but not with physical activity levels. Renovations have limited the potential to increase physical activity until factors influencing usage and physical activity behavior are better understood.

## 1. Introduction

Regular moderate-intensity physical activity (PA) can confer substantial health benefits for children, including reducing risks for chronic diseases, maintaining healthy body weight, and improving social and mental health [1]. Despite the importance of PA to health, only about one-quarter of the Australian children meet the recommended 60 minutes of moderate to vigorous physical activity (MVPA) daily [2]. Inactive children are at greater risk of being overweight or obese [3–5]. In Australia, about one-quarter of children are either overweight or obese [6, 7].

Structured as well as unstructured activities like walking, cycling, and in particular outdoor active free play have been shown to be associated with children's PA [8, 9]. Neighborhood environments [10] as well as specific aspects, such as park quality and proximity to playgrounds, have also been shown to be associated with numerous psychological and social benefits to adults and children [11, 12] and healthy body mass index in Canadian children [13]. Public parks can also provide recreational spaces for active play to support children's PA participation [12, 14–16].

Among Australian schoolchildren, time spent outdoors is correlated to time spent in MVPA and can predict higher MVPA levels in boys [8]. However, children are more likely to spend outdoor time in home backyards than in playgrounds [17]. To support opportunities for children's PA, particularly in low-income urban environments in which homes have small or no backyards, the provision of quality public outdoor spaces which support PA may be an important public health strategy. Among recreational areas specifically designed to support children's PA, such as playgrounds, swimming pools and playfields, playgrounds are the most frequently used [18]. Also, children are more active in playgrounds than in other park activity areas such as fields [19].

Cross-sectional and qualitative studies of park and playground use by children identified perceptions of quantity and quality of park amenities [20, 21] and park and neighborhood safety to be linked to parents encouraging their child to use playgrounds [13, 16, 20–24] and parks [25]. These studies also highlighted that the distance to parks, which is the strongest determinant of adults' visits [14, 26], is less clear in children's park use and may be secondary to park amenities [22, 23].

Environmental interventions such as park upgrades have the potential to facilitate increased park usage and to provide more opportunities for PA. In school settings, playground improvements increase student's playground usage and PA [27–32]; yet the impact of playground renovations in the community on usage and PA is still unclear. Quigg and colleagues [33] found environmental changes alone to be ineffective in increasing PA. Total daily PA was not significantly different at followup among school-age children living in a community with renovated playgrounds compared to those with un-renovated playgrounds. These results need to be interpreted with caution as only two of six playgrounds in the intervention setting actually underwent renovations, playground usage was not measured, and the type of playgrounds (i.e., “standalone” or within a park) was not described. Tester and Baker [34] found increased visits in a renovated playground among girls but a decline among boys. Thus, the effects of structural changes alone on children's playground usage and PA levels remain unknown.

An opportunity to observe the effects of environmental changes in a children's playground on usage, PA behavior, and users' perceptions arose in a metropolitan area of Sydney, Australia. Using a quasi-experimental design, the aims of the current study were to (1) determine if an urban park renovation that included playground alterations affects usage and PA in children within playgrounds; (2) determine whether playground alterations affects parents' self-report of playground visitation post intervention, in association with proxy reports of children's PA levels; and (3) assess parental impressions of environmental features (e.g., equipment safety) of the renovated playground post intervention.

## 2. Methods

### 2.1. Study Setting

The intervention Park A for this study is located in a lower socioeconomic urban neighborhood within the city of Sydney, the council responsible for the central Sydney areas. The comparison park (Park B) was chosen for its similarities to Park A and is located in a nearby urban neighborhood within the city of Sydney. These similarly sized parks each included a playground, a large open area, and a sports field; several small “standalone” playgrounds can be found within a short walk of each of these parks. The neighborhood population of Park B is generally socioeconomically similar to Park A (e.g., percent population identified as Indigenous) though it has a higher employment rate and a higher proportion of English-only speaking households [35, 36].

### 2.2. Elements of Park Upgrade

Sydney's recreational strategy aims to “increase participation by residents in physical activity thereby enhancing their health and well-being” [37] and its objectives for this park renovation included provision for recreation and children's play by “amenities to facilitate use and enjoyment …including but not limited to children's play equipment” [38]. Specific changes in the park renovation included upgrading paths and adding new greenery, lighting, and facilities (e.g., park furniture). More green space was created by opening the adjacent sports field to public use, thus increasing the accessible park size from 2.2 to 4.6 ha.

**Figure 1:**
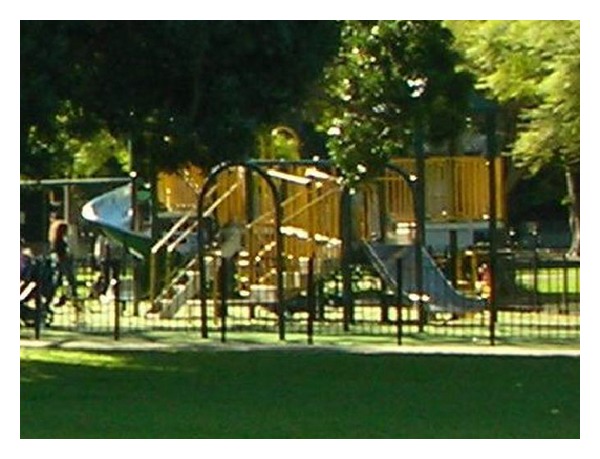
Playground in Park A, prerenovation. The gated playground included a large multifunction apparatus on soft-fall flooring in a gated area in the middle of the park.

**Figure 2:**
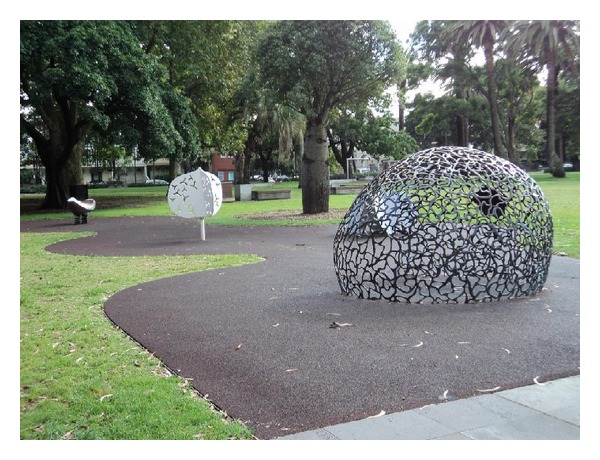
Playground in Park A, postrenovation. Play sculptures on soft-fall flooring were installed in an open area.

Prior to the upgrade, there was one playground located near the center of the park. As shown in Figure 1, the gated playground had soft-fall flooring and included a large multifunction apparatus, multiple swings, slides, and other equipment. The new children's playgrounds are three unfenced areas dispersed throughout the park. The playgrounds incorporate the city of Sydney's design mandate to include public art, an aboriginal theme, and, following community consultation, a water play feature. One area includes two swings and a climbing structure designed for use primarily by young children, with wood chips as flooring. For children up to 7 years old, play sculptures with a soft-fall surface and an interactive water feature on cement were installed in a second area, as shown in Figure 2. A third play area, for older children, is comprised of three climbing poles with wood chips as flooring. As part of the upgrade, a large cement area for basketball and skating was installed adjacent to the poles.

**Figure 3:**
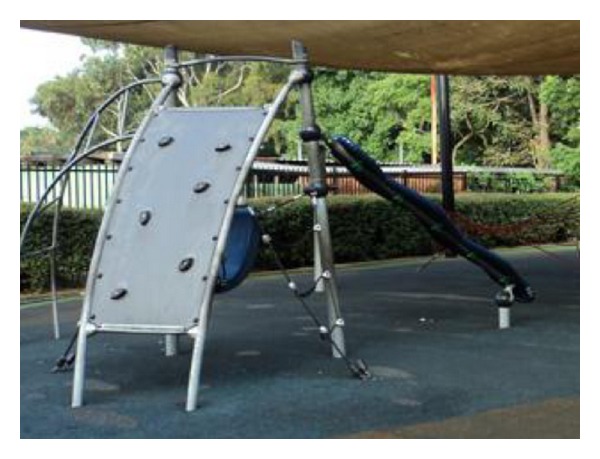
Playground in Park B. The gated and shaded playground featured more traditional style equipment on a soft-fall surface.

The comparison park was chosen for its similar size (4.2 ha) and type of park. Its playground is similar to the prerenovation playground in Park A: a fenced area with soft-fall flooring and containing multifunction apparatuses, swings, slides, and other equipment as shown in Figure 3. The comparison playground had a shade net and equipment pieces were grouped into toddler and school-age sections.

### 2.3. Study Design

This study employed a quasi-experimental pre-post evaluation design with a comparison park. Data from two cross-sectional observational and intercept interviews were collected before and after the upgrade. Data collection involved children aged 2–12 years and their parents or care givers (hereafter referred to as parents) in intervention and comparison parks in May 2007 prior to the upgrade, and nine months after the upgrade completed in May 2009. The study was approved by the University of Sydney Ethics Committee (Ref. no. 04-2007/9905).

### 2.4. Data Collection

#### 2.4.1. Direct Observation

Systematic observations of playground visitors aged 2–12 years were carried out using the System for Observing Play and Recreation in Communities (SOPARC) [31] adapted for use in Sydney [39]. SOPARC uses momentary time sampling to measure park user characteristics and PA behavior and has been determined to produce acceptable reliability and validity [40].

Each park was divided into target areas for observation. The children's playground was one of four preupgrade observation areas in the intervention park. Due to significant changes in park design and accessible area with the upgrade, the postupgrade observation areas were changed to six areas with the three playground areas each being a unique observation area. At analysis, the data from scans for these three playground observation areas were combined as one scan to provide an accurate picture of total playground usage. In the comparison park, the children's playground was one of four observation areas.

Staff scanned target areas from left to right every thirty minutes during the observation periods and recorded park users' gender, age, activity type, and activity intensity. Activity type included both sedentary and active pursuits. Activity intensity was coded light, moderate, or vigorous.

Direct observation was conducted over a 2-week period during clement weather in May 2007 and May 2009. Observations occurred simultaneously at both parks during 2-hour periods in the morning (7:00–9:00), mid-day (11:30–13:30), and afternoon (15:00–17:00). All field staff including the authors (EB) participated in one full day of data collection training, followed by a half-day field practice in park observation. The interrater tests included nine field staff in 2007 and 13 field staff in 2009. The intraclass coefficients (ICC) in 2007 for total counts, gender, age, and PA intensity (light/sedentary versus MVPA) were 0.67, 0.82, 0.94, and 0.68, respectively. The corresponding ICCs for 2009 were 0.89, 0.92, 0.86, and 0.94, respectively.

#### 2.4.2. Park User Interviews

Postupgrade intercept surveys were conducted with consenting park users aged 16 years or older who were accompanied by children under 13 years of age. Ages were verified verbally with participants. Intercept surveys, brief face-to-face interviews with park users, were conducted in both parks using the Sydney Parks User Interview Survey [39] to measure parent's demographics, PA behavior, and park usage. Postintervention interviews were supplemented with a retrospective question on playground use to capture the intervention context and questions regarding children's playground usage. Perceptions of park features were measured using a 5-point Likert scale, and playground features used a 4-point scale of agreement. These were followed by an open-ended question for additional comments. Respondents were also asked about the PA behavior of their oldest child aged 5–12 years, using questions derived from a state-wide New South Wales Population Health Survey [41]. These questions asked the number of days and hours on weekdays and weekend days a child engaged in PA outside of school hours.

Interviews were conducted by one of the authors (EB) between 10:30 and 17:00 hours on the same days during which direct observation was performed. Interviews were conducted throughout the park space, one interview per target area, rotating through all target areas for each data collection period. In the event of a refusal, another park user within the same area was approached; if no users participated in a given target area, data collection directly continued in the next target area. The researcher (EB) received interview training during the observation training sessions.

Two new pieces of school-age play equipment were installed in Park A in August 2009, and a café in the park grounds opened. Therefore, the intercept survey was repeated on clement days over a two-week period in September 2009.

### 2.5. Statistical Analyses

The primary outcomes were the daily mean number of children visiting playgrounds, and the proportion of children engaging in MVPA based on observational data. The first set of analyses examined both playground use and MVPA. Using data from the Bureau of Meteorology, dates from pre- and postintervention were matched for clemency; this resulted in data from five weekdays and two weekend days from each time period being used. Data points concerning infants were omitted. Due to large fluctuations in usage, there was some variation in the number of scans per 2-hour observation periods both within and between parks. To standardize this difference, usage means (observed persons per observation period) were calculated for playground usage for total children and by gender. Overall differences between pre- and postintervention counts of children and children engaged in MVPA were tested using a generalized linear model. An initial examination of the data revealed that both counts were not normally distributed and too dispersed for a Poisson model, so these were modeled with a negative binomial distribution (with log link function) to allow for overdispersion. Deviance (from the “perfect” model) was used to assess the fit of the model. Models were fitted for each count (total children + total in MVPA) with park (Park A versus Park B) and time (pre- and postintervention) and adjusted for gender (as a covariate). The interaction between the park and time variables was used to test if the amount of change within parks from pre- to postintervention was meaningfully different between Park A and Park B.

The second set of analyses examined parent's playground usage profile, their familiarity with Park A's playground prior to renovation, and parent's proxy report of their children's PA levels, based on intercept survey. Survey participation in Park B (*n* = 17 parents) was inadequate for analysis and therefore is not presented here. Chi-square tests were used to test associations between parent usage profile and follow-up study groups at significance level of 0.05. The questions and computation of children's PA levels followed the protocol from the New South Wales Population Health Survey [41]. Parents reported the number of hours on weekdays and weekend days their oldest child between the ages of 5 and 12 years engaged in physical activity outside of school hours. Total physical activity hours per week were summed, and a daily mean was calculated. Sufficient physical activity was defined as meeting or exceeding the recommended daily hour of at least moderate-intensity physical activity. 

The third set of analyses examined parent's impressions of environmental features (e.g., equipment safety) of the renovated playground, based on intercept survey. For open-ended questions, answers were grouped according to themes. Statistical analyses were conducted using PASW 19 (IBM-SPSS Inc., 2009) and SAS 9.2 (SAS Institute, Cary, NC, USA).

## 3. Results

### 3.1. Playground Use

As shown in Table 1, children's playground usage at baseline was lower in the intervention Park A compared to comparison Park B (likelihood ratio test: *P* = 0.03). This difference could be due to a childcare facility being located next to the comparison park. However, there was no detectable difference between the parks at followup (interaction between park and time: *P* = 0.42), when the mean number of children in the playground in Park A increased by approximately 10% (*P* = 0.74) and decreased by 22% at Park B (*P* = 0.42). There was no difference in usage according to gender (*P* = 0.97) in the use of parks before or after intervention. When analyzed by gender, the playgrounds exhibited similar changes in usage over time in girls but not in boys. Attendance decreased for girls at both parks, but for boys this remained unchanged (Park B) or increased (Park A); however, none of these pre-post renovation changes were statistically significant.

At baseline, fewer children performed MVPA in Park A than in Park B (*P* = 0.02), as shown in Table 2. After the park upgrade, there was no detectable difference between parks in the number of children engaged in MVPA (interaction between park and time: *P* = 0.73); the proportion of physically active children had decreased by 41% at the intervention playground and by 32% at the comparison playground. Boys were slightly more active than girls in both parks at baseline and followup although this difference was not statistically significant (*P* = 0.09). Within the intervention park, there was a significant decline in girls engaging in MVPA at followup (*P* = 0.04).

### 3.2. Parental Park Use and Physical Activity Level of Children

A total of 140 parents (73% response rate) took part in the postintervention interviews. There were no significant differences in sociodemographic characteristics between the survey participants at the two survey points (*n* = 75 in May and *n* = 65 in September), with the exception of a higher percentage of mothers in September (73.0%) than in May (53.2%, *P* < 0.01). 

More than half of the parents visited Park A at least once per week (Table 3). First time visitors accounted for approximately 13% of the respondents. A lower proportion of survey respondents from September had visited the playground before the renovation (49.2%) than those from May (66.7%, *P* = 0.04). Respondents reported that at least half of the children achieved at least one hour daily PA (Table 4). There was no significant difference with time or by gender. Attainment of sufficient daily PA was not correlated with frequency of park use (*P* = 0.23).

### 3.3. Park Features

Parents were overwhelmingly positive about the renovated Park A, with 88% agreeing it was well-maintained and 75% finding it attractive. General park safety was rated positively by 68% of parents; of those feeling unsafe, crime (40.4%) and safety (34%) in the park were the main concerns. The majority (61%) felt the park offered social opportunities. Opinions regarding the play equipment characteristics were also mostly positive. Most parents agreed there was a good variety of equipment (64.5%), the equipment was safe and in good condition (83.9%), and it was adventurous or exciting (71.9%). Participants were divided on whether there was sufficient play equipment (49.6% agreement). In general, perceptions of the park and playground were not affected by time of data collection (May versus September; *P* > 0.1 for all variables) or frequency of playground visitation (*P* > 0.41). The two exceptions were that the less frequent (less than once per week) playground visitors were more likely than high frequency (at least once per week) visitors to find the equipment adventurous or exciting (*P* < 0.03) and safe and in good condition (*P* < 0.03).

The majority (69%) of interviewed parents offered one or more remarks when asked for comments regarding the upgraded playground; sociodemographic differences between these parents and the total sample were not statistically significant (*P* > 0.07). Two major themes emerged from these comments: a lack of play equipment and a presence of safety concerns. Forty-nine respondents believed that the playground lacked certain playground equipment, particularly slides and sufficient swings. Comments regarding safety related primarily to concerns about the unfenced toddler play area: its placing close to a road, its lack of fencing, and its woodchip surfacing. Comments regarding the nontraditional play equipment comprised a third theme. Parent's perceptions were mixed, describing these structures as “an art gallery,” “style over substance,” and “of limited value to kids.” Participants' main reason to visit playgrounds was to let children play, and nearly all parents (90%) reported frequenting other playgrounds as well. Their main reason(s) for choosing the other playgrounds included equipment and convenience. Playgrounds which offered a variety of equipment were popular; the amount or type of equipment was important as well. Typical comments included choosing a playground because it offered “lots of equipments,” “lots of swings,” or “big slides.”

## 4. Discussion

While increasing children's PA levels may not be the primary motivation for playground/park upgrades by local council authorities, cross-sectional evidence to date suggests that using public open spaces involves some level of physical activity. Examining the level of effects following physical environmental changes is, therefore, worth investigating. This evaluation study of a natural experiment provides preliminary evidence on the impact of a playground upgrade on both usage and PA behaviors among children. We found that following playground renovation usage between the intervention and comparison parks did not differ. While usage among girls decreased at both playgrounds, the (nonsignificant) decline was less marked at the renovated playground. Among boys, usage increased more at the renovated playground, but this was also nonsignificant. This finding is consistent with other studies which found more boys than girls using renovated school playgrounds [28] and at renovated public playfields [34]. Together, these results suggest that structural changes at public playgrounds can influence boys' usage but may be less effective in affecting usage by girls.

This study observed a decline in children's MVPA levels post renovation; this decrease was significant in girls at the renovated playground. Other playground renovation studies have found increases [27, 42] or no difference [28] in activity levels. The conflicting results of those studies with present results may be explained by differences in study setting (school playgrounds) and design. The changes in the playground design which dispersed the play equipment throughout the renovated park likely also impacted the MVPA levels: the resulting playground scan areas now also included amenities (e.g., picnic tables and large open spaces) associated with more sedentary behavior [29, 43, 44].

A unique contribution of the present study is the interviews which were conducted simultaneously with the observational measurements. These interviews elucidate factors which may explain the observed lack of significant positive effect in playground usage and MVPA. We found that although the perception of overall park safety during the day was not related to frequency of usage (*P* = 0.49), perception of the playground equipment safety was a factor, with frequent visitors less inclined to perceive the equipment as safe and in good condition than infrequent visitors (*P* < 0.03). Specific concerns expressed by parents in regards to the safety of fencing and flooring of the renovated playground suggest these safety factors could play a role in the observed usage and MVPA levels in that specific area. Parents are hesitant to endanger children through use of playground equipment perceived as dangerous [45]. If these parents discouraged their children from using the “unsafe” equipment, then the children are left to play in empty spaces, and in such areas children are less active than in areas with play structures [29].

The perception of equipment as “adventurous or exciting” may also be important to stimulate playground usage, at least initially. In this study, there was a trend for increased usage and an increase in the proportion of parents who were new to the renovated playground after additional equipment was added, but a lower proportion of frequent users, compared to infrequent users, agreeing that the equipment was “adventurous or exciting” in character (chi-square test with *P* < 0.03). Thus, the equipment appears to have stimulated attraction but not to play a role in continued playground use. This agrees with a recent study [46] which found unique equipment merely fosters interest in, but not usage of, public playgrounds.

For sustainable and active use, specific types of equipment (e.g., water features and maneuverable equipment) appear to be important. Though parents found the water feature on this renovated playground attractive, what influenced them to visit other local playgrounds was the presence of traditional equipment, like swings or slides, or maneuverable materials like play trucks. Previous studies agree that specific types of equipment (e.g. water features and loose equipment) influence public and school playground use [16, 21, 22, 29]. In this study, the sculptures supported creative play but, by design, were focused on passive use (e.g. sitting and reading) [47], as opposed to providing for a variety of physical activities like hanging and sliding; they were “aesthetic but not functional”. Thus, the sculptures' focus possibly explains the observed decline in MVPA in the study playground.

If equipment qualities like safety or functionality are important for supporting playground usage and PA behaviors, could the amount or variety of equipment also play a role? At first glance, usage appears to depend on equipment amount but not variety. In this study, observed usage slightly increased on the renovated playground which had more pieces of equipment but offered less variation in activities compared to preupgrade. This is consistent with a previous finding [48] that usage in renovated playgrounds was related to the number of equipment pieces but not the number of unique types of play offered. However, parent comments in this study and others [16, 21, 22] support the ideas that specific equipment types influence playground usage and diversity of equipment is desirable [23, 49]. Consider also that perceptions of equipment sufficiency and variety in this study remained unchanged following the additions of the basketball courts and skate park. This equipment was designed for older children but tended to be used more by adolescents and young adults and hence may have been perceived by the parents as having no play value. Thus, both the amounts of equipment and perceived play value may be factors in playground usage.

Any relationship between MVPA behavior and the amount or variety of equipment may also have underlying nuances. In this study, MVPA decreased in the renovated playground which had more equipment but less play variety. Previous studies have reported that children's PA levels were directly correlated to the number of equipment pieces on school playgrounds [50, 51]; however, MVPA levels were not correlated with the total number or number of unique equipment types in a separate study [48]. The key to supporting children's PA attainment may instead relate to functional aspects of the play equipment (play affordances) [29, 49]. Additionally, these affordances may vary with gender in terms of preferred equipment, types of play [27, 45, 52], and PA levels: girls' PA levels were higher in renovated playground areas with swings, play equipment, and fields and lower in renovated hard surface areas such as basketball courts than in control playgrounds [27]. In this study, the observed decrease in girls' MVPA levels following the renovation which reduced the number of swings and other active play equipment supports the idea that specific play affordances are critical to fostering PA, particularly in girls.

Further, this study found no correlation between playground usage and the level of children's daily PA. Other research noted that children obtain less than 2% of their PA in public parks [53]. This can mean that either playground use does not lead to overall daily PA levels among children, or that more could be done to maximize playgrounds (and parks) as an affordable setting to get children to be more active, particularly in low-income neighborhoods.

There are a number of limitations in this study. Firstly, the generalizability is limited because the findings relate to one intervention and comparison park. Secondly, changes in playground layout resulted in observation scan areas at followup that include both play equipment and other park amenities. This complicated the comparison of playground usage and PA levels. While the observation periods occurred at predetermined times throughout the day totaling six hours per day for 14 days, this period may neither be representative of total playground use nor capture secular variations in usage and PA. Our inter-rater agreement for PA levels (sedentary/light versus MVPA) was lower than that reported in a previous study which used a similar tool [40], but also higher at followup. This could be because more people were doing light/sedentary activities at followup; there were relatively few PA observations classified at MVPA level. Alternatively, there may be a greater error in our baseline survey in relation to MVPA assessment, and so our findings for decline may be biased. Furthermore, this was a short-term followup post renovation; a long-term followup might result in increased usage or other changes. The study estimates, however, do provide a snapshot of playground use by gender and PA level.

In addition, no intercept survey of parents was conducted prior to the upgrade, thus limiting the ability to infer change from baseline to followup. Due to the small sample size of those completing intercept surveys in the comparison park, we were unable to compare parental views between intervention and comparison parks. The intercept surveys relied on parental proxy reports of their children's PA, which could result in biased reporting.

Despite its limitations, this study contributes some initial evidence on the relationship between the built environment and PA of children, in particular examining children's usage of and PA levels in playgrounds following an upgrade to an inner city park in Sydney. In addition, this study presents findings of parental preferences for play equipment and playground design in the same areas and at the same time as the direct observation of the children was conducted.

## 5. Conclusions

In this study playground renovations did not translate to an increase in observed MVPA among children. Parental perceptions of playground equipment may explain attendance and PA levels. This study adds to the growing evidence that there are many factors influencing playground usage and PA behavior. Future research should investigate the relationship between specific play equipment characteristics, children's usage, and PA behavior in playgrounds so that the health benefits of environmental changes are maximized. Determining whether such relationships are influenced by playground setting (e.g. in school or in public parks) and other features (e.g. presence of other activity areas) can help guide policies which promote sustained active usage of playgrounds.

## Figures and Tables

**Table 1:** Children's playground usage in intervention Park A and comparison Park B, pre- and postintervention, expressed as mean number of children per 2-hour observation period.

	Boy		Girl		Total children	
	Pre^a^	Post	Pre^a^	Post	Pre^a^	Post
Intervention Park A*	3.95 (4.68)	5.33 (5.92)	5.05 (5.41)	4.62 (6.30)	4.50 (5.03)	4.98 (6.05)
Comparison Park B	7.76 (8.30)	7.71 (6.91)	9.29 (11.59)	5.67 (6.37)	8.52 (9.99)	6.69 (6.64)

Note: Data included observations from scans performed every 30 minutes during three 2-hour periods on 5 weekdays and 2 weekend days; *n* = 84 scans for Park A and Park B (preintervention), and 80 for Park B post intervention. *In Park A at followup, three scan areas included play equipment; these three scans were combined as one for comparison purposes. Means were compared via a generalized linear model, with significance set at *P* < 0.05. ^a^A significant difference was found between parks.

**Table 2:** Children engaged in MVPA in playground in Intervention Park A and Comparison Park B, preand post intervention, expressed as mean number of children engaged in MVPA per 2-hour observation period.

	Boy		Girl		Total children	
	Pre^a^	Post^a^	Pre^a^	Post^a,b^	Pre^a^	Post^a^
Intervention Park A*	1.19 (2.09)	1.10 (1.51)	1.14 (2.37)	0.24 (0.44)	1.17 (2.21)	0.67 (1.18)
Comparison Park B	3.19 (4.76)	2.38 (3.79)	2.52 (3.03)	1.57 (2.04)	2.86 (3.95)	1.98 (3.03)

Note: Data included observations from scans performed every 30 minutes during three 2-hour periods on 5 weekdays and 2 weekend days; *n* = 84 scans for Park A and Park B (preintervention), and 80 for Park B postintervention. *In Park A at followup, three scan areas included play equipment; these three scans were combined as one for comparison purposes. Means were compared via generalized linear model, with significance set at *P* < 0.05. ^a^A significant difference was found between parks. ^b^A significant difference was found between pre and postintervention MVPA for girls in Park A.

**Table 3:** Playground use profile of parents in playground of intervention Park A as measured by intercept survey postintervention, percent (number).

	Total (*n* = 140)	May (*n* = 75)	September (*n* = 65)	Chi-square (*P* value)
Playground visit frequency				
At least once per week	59.4 (79)	57.7 (41)	61.3 (38)	*χ* ^2^_(2)_ = 1.51 (0.47)
1-2 per fortnight or less	27.1 (36)	31.0 (22)	22.6 (14)	
First time	13.5 (18)	11.3 (8)	16.1 (10)	
Visited playground before renovation				
Yes	58.6 (82)	66.7 (50)	49.2 (32)	*χ* ^2^_(1)_ = 4.36 (0.04)
No	41.4 (58)	33.3 (25)	50.8 (33)	

**Table 4:** Physical activity level of children of playground intervention Park A users as measured by parental proxy questionnaire, postintervention.

Physical activity	Total % (*n* = 58)	May % (*n* = 34)	September % (*n* = 24)	Chi-square (*P* value)
Sufficient activity	55.2 (32)	58.8 (20)	50.0 (12)	*χ* ^2^_(1)_ = 0.44 (0.51)
Insufficient activity	44.8 (26)	41.2 (14)	50.0 (12)	

Parents were asked to report the number of hours of physical activity on weekdays and weekend days engaged in by their oldest child between the ages of 5 and 12 years. A daily mean of MVPA was then calculated. Sufficient physical activity was defined as attaining the recommended daily hour of MVPA.
